# The Impact of Self- and Relation-Inferred Efficacy on Physical Activity in Older Adult Couples

**DOI:** 10.1093/geroni/igab046.2789

**Published:** 2021-12-17

**Authors:** Tiana Broen, Yoonseok Choi, Denis Gerstorf, Maureen Ashe, Kenneth Madden, Christiane Hoppmann

**Affiliations:** 1 University of British Columbia, Vancouver, British Columbia, Canada; 2 Humboldt University of Berlin, Humboldt University of Berlin, Brandenburg, Germany; 3 University of British Columbia, University of British Columbia, British Columbia, Canada

## Abstract

Previous research has indicated that physical activity (PA) is a health-promoting behavior that is closely linked in couples. However, few studies have examined how PA is intertwined among couples in their everyday lives. For example, relation-inferred efficacy (RIE) is an individual perception that captures whether a close other believes in one’s own abilities to perform specific behaviours; it originates from the sports literature on coach-athlete dyads and has been shown to shape athlete performance. Applying a repeated daily life assessment design, the current study targets self-efficacy (SE) and relation-inferred efficacy (RIE) as predictors of PA in older adult couples, as well as potential moderators when obstacles occur. We hypothesized that: (a.) There is a main effect of SE and RIE on PA. (b.) PA is lower on days when people anticipate barriers (c.) SE and RIE moderate the time-varying relationship between PA and barriers. Heterosexual couples (N=108 couples, Mage=70.5 years, SD=6.70) rated their SE and REI, completed daily electronic questionnaires asking about barriers and wore an accelerometer to capture indices of PA across seven days. In line with past work, SE (r(2438)=.13, p=<.001) and RIE (r(2438)=.14, p=<.001) were significantly related to total moderate-to-vigorous PA (MVPA) and step counts. A series of multilevel models were fit to examine the hypotheses. Preliminary analyses indicated that RIE (estimate=3.93, SE=1.49, p=.009) is a stronger predictor of MVPA than SE (estimate=2.83, SE=2.02, p=.16). Further analysis will be conducted to unpack daily life circumstances that create barriers to PA, including daily pain, anxiety, and tiredness.

